# Induction of labour versus expectant monitoring for gestational hypertension or mild pre-eclampsia between 34 and 37 weeks' gestation (HYPITAT-II): a multicentre, open-label randomised controlled trial

**DOI:** 10.1186/1471-2393-11-50

**Published:** 2011-07-07

**Authors:** Josje Langenveld, Kim Broekhuijsen, Gert-Jan van Baaren, Maria G van Pampus, Anton H van Kaam, Henk Groen, Martina Porath, Martijn A Oudijk, Kitty W Bloemenkamp, Christianne J de Groot, Erik van Beek, Marloes E van Huizen, Herman P Oosterbaan, Christine Willekes, Ella J Wijnen-Duvekot, Maureen T M Franssen, Denise A M Perquin, Jan M J Sporken, Mallory D Woiski, Henk A Bremer, Dimitri N M Papatsonis, Jozien T J Brons, Mesruwe Kaplan, Bas W A Nij Bijvanck, Ben-Willen J Mol

**Affiliations:** 1 Department of Obstetrics and Gynecology, Maastricht University Medical Centre. GROW - School for Oncology and Developmental Biology, The Netherlands; 2 Department of Obstetrics and Gynecology, Martini hospital, Groningen, The Netherlands; 3 Department of Obstetrics and Gynecology, Academic Medical Centre, Amsterdam, The Netherlands; 4 Department of Obstetrics and Gynecology, Onze Lieve Vrouwe Gasthuis Amsterdam; 5 Department of Neonatology, Emma Children's Hospital, Academic Medical Centre, Amsterdam, The Netherlands; 6 Department of Epidemiology, University Medical Centre Groningen, The Netherlands; 7 Department of Obstetrics and Gynecology, Maxima Medical Centre, Veldhoven, The Netherlands; 8 Department of Obstetrics and Gynecology, University Medical Centre Utrecht, The Netherlands; 9 Department of Obstetrics and Gynecology, Leiden University Medical Centre, The Netherlands; 10 Department of Obstetrics and Gynecology, VU Medical Centre Amsterdam, The Netherlands; 11 Department of Obstetrics and Gynecology, St. Antonius Hospital Nieuwegein, The Netherlands; 12 Department of Obstetrics and Gynecology, Haga Hospital Den Haag, The Netherlands; 13 Department of Obstetrics and Gynecology, Jeroen Bosch Hospital, s’-Hertogenbosch, The Netherlands; 14 Department of Obstetrics and Gynecology, VieCuri Medical Centre, Venlo, The Netherlands; 15 Department of Obstetrics and Gynecology, Medical Centre Leeuwarden, Leeuwarden, The Netherlands; 16 Department of Obstetrics and Gynecology, Canisius-Wilhelmina Hospital, Nijmegen, The Netherlands; 17 Department of Obstetrics and Gynecology, Radboud Univeristy Nijmegen, Nijmegen, The Netherlands; 18 Department of Obstetrics and Gynecology, Reinier de Graaf Hospital, Delft, The Netherlands; 19 Department of Obstetrics and Gynecology, Amphia Hospital Breda, The Netherlands; 20 Department of Obstetrics and Gynecology, Medical Spectrum Enschede, The Netherlands; 21 Department of Obstetrics and Gynecology, Saxenburgh Group, Hardenberg, The Netherlands; 22 Department of Obstetrics and Gynecology, Isala Hospital, Zwolle, The Netherlands

## Abstract

**Background:**

Gestational hypertension (GH) and pre-eclampsia (PE) can result in severe complications such as eclampsia, placental abruption, syndrome of Hemolysis, Elevated Liver enzymes and Low Platelets (HELLP) and ultimately even neonatal or maternal death. We recently showed that in women with GH or mild PE at term induction of labour reduces both high risk situations for mothers as well as the caesarean section rate. In view of this knowledge, one can raise the question whether women with severe hypertension, pre-eclampsia or deterioration chronic hypertension between 34 and 37 weeks of gestation should be delivered or monitored expectantly. Induction of labour might prevent maternal complications. However, induction of labour in late pre-term pregnancy might increase neonatal morbidity and mortality compared with delivery at term.

**Methods/Design:**

Pregnant women with severe gestational hypertension, mild pre-eclampsia or deteriorating chronic hypertension at a gestational age between 34^+0 ^and 36^+6 ^weeks will be asked to participate in a multi-centre randomised controlled trial. Women will be randomised to either induction of labour or expectant monitoring. In the expectant monitoring arm, women will be induced only when the maternal or fetal condition detoriates or at 37^+0 ^weeks of gestation. The primary outcome measure is a composite endpoint of maternal mortality, severe maternal complications (eclampsia, HELLP syndrome, pulmonary oedema and thromboembolic disease) and progression to severe pre-eclampsia. Secondary outcomes measures are respiratory distress syndrome (RDS), neonatal morbidity and mortality, caesarean section and vaginal instrumental delivery rates, maternal quality of life and costs. Analysis will be intention to treat. The power calculation is based on an expectant reduction of the maternal composite endpoint from 5% to 1% for an expected increase in neonatal RDS from 1% at 37 weeks to 10% at 34 weeks. This implies that 680 women have to be randomised.

**Discussion:**

This trial will provide insight as to whether in women with hypertensive disorders late pre-term, induction of labour is an effective treatment to prevent severe maternal complications without compromising the neonatal morbidity.

**Trial Registration:**

NTR1792 Clinical trial registration: http://www.trialregister.nl

## Background

Hypertensive disorders (including gestational hypertension (GH) and pre-eclampsia (PE)) complicate 10% of all pregnancies and can result in severe complications for the mother such as eclampsia, placental abruption, pre-term delivery, the syndrome of Hemolysis, Elevated Liver enzymes and Low Platelets (HELLP) and ultimately even neonatal and maternal death [1]. Hypertensive disorders in pregnancy are the largest cause of maternal mortality in the Netherlands [2,3]. Unfortunately, maternal mortality in the Netherlands has increased over the past decade, with pre-eclampsia remaining the number one cause of maternal death [4].

The majority of the cases of GH and PE declare itself after 32 weeks. As the origins of hypertensive disorders are probably related to faulty implantation of the placenta early in pregnancy, the only causal treatment of the disease is immediate delivery. Delivery will result in removal of the placenta, which will result in disappearance of the signs and symptoms of GH or PE within a few days. The timing of induction of labour has been debated, as induction was thought to increase the number of caesarean sections and in case of pre-term delivery, might compromise neonatal outcome. Our recent HYPITAT-I study provided us evidence that in women with GH and PE above a gestational age of 37 weeks, induction of labour not only resulted in a decrease of progression to severe pre-eclampsia or maternal complications, but unexpected also in a decrease in the number of caesarean sections (19% versus 14%, RR 0.75, 95% CI 0.55 - 1.04) [5]. Moreover, induction of labour showed a trend to a better neonatal outcome, a better maternal quality of life and a reduction in costs [6,7]. However for women with GH, PE or deteriorating chronic hypertension in the late pre-term period (e.g. 34, 35 and 36 weeks' gestation), there remains uncertainty on the best policy. The situation in these patients is different, as apart from maternal morbidity, the condition of the child is at state. Late pre-term born children experience significantly more morbidity, compared to children born at term. Unfortunately, children born from mothers with a hypertensive disorder were mostly excluded from studies [8-10]. Thus, for women with hypertensive related complications of pregnancy between 34 and 37 weeks, the issue on whether these women should be monitored expectantly or not, remains an obstetric dilemma. In addition, the NICE guideline "hypertensive disorders during pregnancy" refers in the consensus statement to the issue of mild or moderate pre-eclampsia between 34 and 36 weeks of gestation in terms of a "grey zone" at which the optimal timing of birth is not clear and more research is necessary for this period [11].

In view of the relatively poor outcome of women with hypertensive pregnancy complications in The Netherlands as compared to abroad and in view of the neonatal morbidity in case of a late pre-term birth, there is a need for critical evaluation of the management of hypertensive diseases during pregnancy in the late pre-term period in the Netherlands.

With lack of good clinical evidence on the subject and the resulting practice variation, we think that additional data comparing immediate delivery and expectant monitoring in patients with hypertensive disease late pre-term are urgently needed. We therefore propose a nationwide randomised clinical trial and an economic cost analysis as well as quality of life analysis on the subject. We aim to provide data on the balance between cost (both financial and in terms of neonatal morbidity) and effects (prevention of maternal complications).

## Methods/design

### Aims

The aim of this study is to investigate whether planned induction of labour compared to expectant monitoring in women with gestational hypertension, mild pre-eclampsia or deteriorating chronic hypertension at gestational age of 34 - 37 weeks of pregnancy, will reduce maternal morbidity and/or increase neonatal morbidity. We hypothesize that induction of labour will reduce maternal morbidity and mortality, but to the costs of increased neonatal morbidity. The study will also provide insight on whether induction of labour in women with GH, PE or deteriorating chronic hypertension in the late pre-term period will influence the quality of life and costs compared to expectant monitoring. Long term follow-up of the neonates and the mothers will be formulated.

### Participant/Eligibility Criteria

Patients 18 years of age or older are eligible if they have gestational hypertension, mild pre-eclampsia or deteriorating chronic hypertension at a gestational age between 34^+0 ^and 36^+6 ^weeks of pregnancy. The diagnosis of GH is made in case the diastolic blood pressure is equal to or above 100 mmHg at two occasions at least six hours apart in a woman who was normotensive until at least 20 weeks of gestation. The diagnosis of mild PE is made in case the diastolic blood pressure is above 90 mmHg at two occasions at least six hours apart in a woman who was normotensive until at least 20 weeks of gestation and if proteinuria exists (> 300 mg total protein in a 24 hour urine collection or > 30 in a spot urine protein:creatinine ratio). The diagnose of chronic hypertension is made in case the diastolic blood pressure is equal to or above 90 mmHg at two occasions at least six hours apart, diagnosed before 20 weeks of gestation. Women with chronic hypertension are eligible in case there is need for additional medication or in case superimposed PE is diagnosed between 34^+0 ^and 36^+6 ^weeks' gestation.

Patients with singleton or multiple pregnancies are eligible, independent of the position of the fetus (i.e. cephalic or breech). Neither diabetes mellitus, nor small for gestational age nor a history of caesarean section are exclusion criteria. Randomisation only occurs after informed consent.

Exclusion criteria are a diastolic blood pressure equal to/greater than 110 mmHg despite medication, systolic blood pressure equal to/greater than 170 mmHg despite medication, proteinuria equal to/greater than 5 g/L, eclampsia, HELLP syndrome, pulmonary oedema or cyanosis, oliguria less than 500 ml in 24 hours, renal disease, heart disease, HIV-positive, non-reassuring fetal heart rate, zero-flow or reverse flow in the umbilical artery, fetal abnormalities including abnormal karyotype, severe pre-eclamptic complaints such as frontal headache or ruptured membranes.

### Procedures, recruitment, randomisation and collection of baseline data

Eligible women will be identified by the local research coordinator and/or the staff of participating hospitals. After counselling and reading the patient information form, patients will be asked for written consent. We will provide patient information in Dutch as well as several other languages (Turkish, French, Spanish and English), in order to facilitate inclusion of patients from cultural minorities. After informed consent, patient data will be entered in a web-based database, which will also facilitate randomisation.

Prior to randomisation, we will perform a digital examination to establish a Bishop score and we will measure cervical length. Women will be randomly allocated to either delivery within 24 hours (experimental arm) or expectant monitoring (control arm) until 37 weeks of gestational age. The study will be an open label study, as it is impossible to blind the health care workers and patients involved for the strategy to which the woman is allocated. Randomisation will be performed centrally with the use of a permuted-block design, stratified for recruiting centre. Randomisation will be 1:1 for intervention and expectant monitoring management. Although it will not be possible to prevent all cross-overs, both strategies will be performed according to strict criteria, as mentioned below.

At study entry baseline demographic, past obstetric and medical history will be recorded into the web-based Case Report Form (CRF) that is accessible through a closed part of a central website. Details of delivery, maternal and neonatal assessments during pregnancy or post-partum are recorded in the CRF. The collected data will be coded and processed with adequate precautions to ensure patient confidentially.

A subset of 200 women will receive quality of life questionnaires prior to randomisation, one day after randomisation and subsequently after 1 week, 2 weeks, 6 weeks, 3 months and 6 months. Since the gestational age at delivery will differ between the two groups, we will ask the participating women also to complete questionnaires at 1 day and 2 days after delivery. A pain scale will be added to those questionnaires. We hypothesize differences on the emotional and anxiety scales prior to delivery and differences in physical scales after delivery (due to differences in instrumental delivery rates). The questionnaires will contain the Medical Outcome Study 36-Item Short Form Health Survey (SF-36), the European Quality of Life 6 dimensions 3 levels (EuroQoL 6D3L) with subsequent general health Visual Analogue Scale (VAS), the Hospital Anxiety and Depression scale (HADS), and the Symptom Check List (SCL-90), all validated in Dutch and English.

### Interventions

#### Immediately delivery

In the intervention group labour will be induced within 24 hours after randomisation. Induction of labour consists of amniotomy and augmentation of labour by administration of oxytocine. In case of an unfavourable cervix induction of labour will be preceded according to the local protocol. Prostaglandins will not be administered to women with a history of caesarean section. Cervical dilatation in these women will be achieved by a cervical Foley catheter followed by amniotomy and augmentation of labour with oxytocine. In case the patient gives no consent for vaginal delivery (for example breech presentation or history of two caesarean sections) the patient will be delivered by caesarean section within 24 hours after randomisation. After delivery, all patients in the intervention group will be monitored clinically.

#### Expectant monitoring until 37 weeks

In the expectant monitoring group, patients will be monitored until the onset of spontaneous delivery. If onset of labour has not occurred at 37^+0 ^weeks gestation, labour will be induced. Monitoring will consist of assessment of fetal movements as reported by the mother, as well as electronic fetal heart rate monitoring at least twice a week. Maternal evaluation consists primarily of frequent evaluation of blood pressure measurement and screening of urine for protein using a dipstick or protein/creatinin ratio twice a week (24 hour urine collection for protein in case of positive screening). Blood tests (platelet count, liver enzymes and renal function) will be performed in case of abnormal maternal blood pressure and/or proteinuria. In the expectant monitoring group, intervention is recommended in case the fetal or maternal condition does not justify expectant monitoring anymore. These criteria are similar to the exclusion criteria of the trial. All patients in the expectant monitoring group will be monitored clinically until after delivery.

#### Follow-up of women and infants

All details of delivery, maternal and neonatal assessments and admission during pregnancy are recorded in the CRF that is accessible through the website. Mortality and morbidity will be specified for the mother and the child until date of discharge from hospital and six weeks postpartum.

We plan long term follow-up at the age of 2 years with the Ages & Stages Questionnaires (ASQ) and the Child Behavioral Checklist (CBCL). By means of e-mails generated by our web-based registration system, our research nurses will be notified when a child approaches the corrected age of two years. After this alert, the research nurse will approach the parents of the child by phone and announce the sending of the questionnaire. In case the parents do not return the questionnaire, a reminder will be sent. We use the validated Dutch translation of the ASQ, covering the age-range 4-60 months and the validated Dutch translation of the CBCL 11/2 - 5 years [12]. The ASQ is developed as a developmental screener and has been validated to pick up children at risk of a developmental disability. The CBCL (Child Behavioral Checklist) evaluates maladaptive behavioral and emotional problems in children reported by parents. It assesses internalizing (i.e., anxious, depressive, and overcontrolled) and externalizing (i.e., aggressive, hyperactive, noncompliant, and undercontrolled) behaviors. We will compare the total mean scores and T-scores between study arms and in addition we will compare the number of children scoring at 2 standard deviations below the mean of the Dutch reference group. A plan for long term follow-up of the mothers on their cardiovascular risk in the future is in preparation [13].

### Outcome measures

#### Primary outcome measure

The primary outcome measure will be a composite endpoint of maternal mortality, maternal complications (eclampsia, HELLP syndrome, pulmonary oedema, thromoembolic disease) and progression to severe pre-eclampsia. Eclampsia is defined as severe gestational hypertension or pre-eclampsia resulting in maternal seizures [14]. HELLP syndrome is defined as a complication of severe pre-eclampsia involving Hemolysis, Elevated Liver functions, and Low Platelets [14,15]. Thromboembolic disease is defined as deep-vein thrombosis, pulmonary embolism or both. Patients will be examined for deep-vein thrombosis by duplex Doppler if thrombosis is suspected from clinical examination. A diagnosis of pulmonary embolism will be confirmed by pulmonary angiography, computed tomography, magnetic resonance imaging or a ventilation-perfusion lung scan [16-18].

Progression to severe pre-eclampsia is defined as diastolic blood pressure equal/greater than 110 mmHg despite medication, systolic blood pressure equal/greater than 170 mmHg despite medication and/or proteinuria equal to/greater than 5 g/L.

The neonatal primary outcome will be respiratory distress syndrome (RDS). Diagnosis of RDS requires signs of respiratory distress, consistent radiologic features, and oxygen therapy with a fraction of inspired oxygen (FIO2) of 0.40 or greater for at least 24 hours or until death [19].

Secondary outcome measures

Secondary maternal outcomes will be caesarean section rate, instrumental vaginal delivery rate, maternal quality of life and costs. Secondary neonatal outcomes will be transient tachypnoe of the newborn, hypoglycaemia, newborn sepsis, confirmed seizures, necrotizing enterocolitis, hypoxic-ischemic encephalopathy, cardiopulmonary resuscitation or ventilator support within 24 hours after birth, umbilical-cord-blood arterial pH below 7.0, a 5-minute Apgar score of 3 or below, admission to the neonatal intensive care unit (NICU) or neonatal death [19]. Transient tachypnoe of the newborn is defined by the presence of tachypnoe within hours after birth and typical radiologic findings. The diagnosis of necrotizing enterocolitis requires confirmation by radiologic findings, surgery, or autopsy. The diagnosis of hypoglycaemia requires a serum or plasma glucose level of less than 35 mg per decilitre (1.9 mmol per litre) and treatment with intravenous glucose. Newborn sepsis includes both suspected infections (with clinical findings suggesting infection) and proved infections (as confirmed in a subgroup of neonates with positive cultures of blood, cerebrospinal fluid, or urine obtained by catheterization or suprapubic aspiration; cardiovascular collapse; or an unequivocal radiograph confirming infection in a neonate with clinical sepsis).

### Statistical issues

#### Data analysis

Data will be analysed on an intention-to-treat basis. Analysis of data includes comparison of maternal baseline characteristics at randomisation, maternal morbidity and mortality until hospital discharge and 6 weeks post-partum, neonatal morbidity and mortality until hospital discharge, method of delivery, type of hospital care and days of maternal and neonatal hospital stay. Differences between groups with normally distributed data will be tested using the Student's *t *test and for data with a skewed distribution, a non-parametric Mann-Whitney U test will be used. Categorical data will be analysed with χ2 statistics. Calculation of the percentages will be based on the number of valid observations. Treatment effect will be presented as relative risk (RR) with 95% CIs, and where appropriate as absolute risk reduction with 95% CIs and number needed to treat. A p-value of less than 0.05 indicates statistically significance. To asses treatment effects between different categories of patients in the trial we will perform subgroup analysis: per gestational age of the fetus per week (34^+0 ^- 34^+6^, 35^+0 ^- 35^+6 ^and 36^+0 ^- 37^+0^), parity (women with versus women without a previous vaginal delivery), hypertensive related disease (gestational hypertension versus pre-eclampsia), the use of corticosteroids (administered prior to delivery versus no use), mode of delivery (vaginal delivery versus caesarean section), caesarean delivery in history (history of a caesarean delivery versus no history of caesarean delivery) and the Bishop score (< 2, 2-6 and > 6).

#### Sample size

We hypothesize that immediate delivery can reduce maternal complications from 5% to 1%. Using a two sided test (alpha error 5%, beta error 20%) and assuming a 10% rate lost to follow-up and protocol violations, we aim to recruit 680 patients. This sample size is also large enough to assess whether there will be relevant differences in the primary neonatal outcome (incidence of RDS around 10% at 34 weeks versus 1-3% at 37 weeks).

#### Economic analysis

We plan an economic analysis alongside the clinical trial. This analysis will be performed from the societal perspective, meaning that we include both medical and non-medical costs to examine the economic impact of both strategies on the whole society.

Within the economic evaluation, we will compare the costs and effects of immediate delivery and expectant monitoring. The key question in the economic evaluation is to assess whether the expected increase of cost of maternal care in case of expectant monitoring will be higher than the expected increase of cost of neonatal care in case of immediate delivery.

The process of care is distinguished into three cost stages (antenatal stage, delivery/childbirth, postnatal stage) and three cost categories (direct medical costs [all health care sector costs], direct non-medical costs [costs outside the health care sector that are affected by health status or health care] and indirect costs of the pregnant woman and her partner [costs of sick leave].

For each stage and each cost category, costs are measured as the volumes of resources used multiplied with appropriate valuation (cost-per-unit estimates, fees, national reference prices). Cost volumes in the antenatal stage consist of admissions of the mother, maternal monitoring with various laboratory tests and fetal monitoring. In this stage, direct non-medical and indirect costs may be generated if role patterns or household routines shift. Costs generated during delivery are dominated by the course of childbirth and type of delivery. Resource utilization in the postnatal stage consist of maternal and neonatal health care during the hospital admission (maternal ward, NICU/neonatology admissions), and primary care following discharge. If neonatal health at discharge is suboptimal, further direct medical, direct non-medical and indirect costs may occur. Hence, for these infants, resource use of infants and/or parents is measured up till the corrected child age of 24 months.

#### Serious adverse events

Serious adverse events will be reported to an independent data safety monitoring committee. A formal interim analysis is not planned.

#### Ethical consideration

This study has been approved by the ethics committee of the Academic medical centre Amsterdam (ref.no MEC 08/244)

#### Confidentially and data security

Initials of participants as well as a local patient number are recorded in the electronic database. Linking names with patients' numbers can only be done in the local clinics. Each participating clinic receives a login name and password to gain access to the web-secured database. The access is restricted to the database of the clinic to which the password and login name belongs. Full access to the entire database is restricted to some members of the research staff.

## Discussion

Gestational hypertension (including chronic hypertension) and pre-eclampsia are important hypertensive disorders during pregnancy which are associated with increased maternal and neonatal morbidity and mortality. There is no consensus on how to manage these hypertensive disorders between 34 and 37 weeks 'gestation. Induction of labour will prevent maternal complications, but might also increase the neonatal morbidity due to the pre-term birth.

This trial will provide evidence as to whether or not induction of labour in women with a hypertensive disorder in the late pre-term period is an effective treatment to prevent severe maternal complication, without compromising the neonatal morbidity.

## List of abbreviations used

PE: pre-eclampsia; GH: gestational hypertension; HELLP: Hemolysis, Elevated Liver enzymes and Low Platelets; CRF: Case Report Form; RDS: Respiratory Distress Syndrome.

## Competing interests

The authors declare that they have no competing interests.

## Authors' contributions

MP, MvP, BWM and JL were involved in conception and design of the study. MP, BWM and JL drafted the manuscript. All authors mentioned in the manuscript are member of the HYPITAT-II study group or collaborators. They participated in the design of the study during several meetings and are local investigators at the participating centres. All authors edited the manuscript and read and approved the final draft.

## Pre-publication history

The pre-publication history for this paper can be accessed here:

http://www.biomedcentral.com/1471-2393/11/50/prepub
