# Correction: Induction of labour versus expectant monitoring for gestational hypertension or mild pre-eclampsia between 34 and 37 weeks’ gestation (HYPITAT-II): a multicentre, open-label randomised controlled trial

**DOI:** 10.1186/1471-2393-13-232

**Published:** 2013-12-23

**Authors:** Kim Broekhuijsen, Josje Langenveld, Gert-Jan van Baaren, Mariëlle G van Pampus, Anton H van Kaam, Henk Groen, Martina Porath, Maureen TM Franssen, Ben W Mol

**Affiliations:** 1 Department of Obstetrics and Gynecology, University of Groningen, University Medical Center Groningen, Groningen, The Netherlands; 2 Department of Obstetrics and Gynecology, Atrium Medical Center, Heerlen, The Netherlands; 3 Department of Obstetrics and Gynecology, Academic Medical Center, Amsterdam, The Netherlands; 4 Department Obstetrics and Gynecology, Onze Lieve Vrouwe Gasthuis, Amsterdam, The Netherlands; 5 Department of Epidemiology, University of Groningen, University Medical Center Groningen, Groningen, The Netherlands; 6 Department of Obstetrics and Gynecology, Maxima Medical Center, Veldhoven, The Netherlands

The earliest draft versions of the protocol for our study described the composite adverse maternal outcome as one or more of progression to severe disease, pulmonary edema, thrombo-embolic disease, HELLP syndrome, eclampsia, placental abruption or maternal death. However, there is ongoing debate as to whether progression to severe disease should be considered an adverse maternal outcome [1,2]. Therefore, after obtaining funding which enabled us to increase our sample size to the current sample size of 680, we decided to study a composite adverse maternal outcome excluding progression to severe disease. These changes were incorporated in the protocol as submitted to and approved by the instutional review board;* the current protocol is available from our website (http://www.studies-obsgyn.nl/hypitat2/page.asp?page_id=642). Unfortunately, the change to the maternal outcome definition was not incorporated into the published protocol, which incorrectly includes progression to severe disease in the composite adverse maternal outcome [3].

We also discovered minor differences between the published protocol and the IRB approved protocol. The definition for neonatal morbidity should have contained meconium aspiration syndrome, pneumothorax and/or pneumomediastinum, periventricular leucomalacia, convulsions and other neurological abnormalities. Finally, low 5-minute Apgar score should have been defined as below 7 (as opposed to below 3), and low umbilical artery pH as below 7.05 (as opposed to below 7.0).

These discrepancies were discovered and the correction submitted for publication during recruitment.

* Medical Ethics Committee, Academic Medical Centre, Amsterdam, the Netherlands (ref. 2008/244).

## Pre-publication history

The pre-publication history for this paper can be accessed here:

http://www.biomedcentral.com/1471-2393/13/232/prepub
